# Exercise with food withdrawal at thermoneutrality impacts fuel use, the microbiome, AMPK phosphorylation, muscle fibers, and thyroid hormone levels in rats

**DOI:** 10.14814/phy2.14354

**Published:** 2020-02-07

**Authors:** Antonia Giacco, Giuseppe delli Paoli, Roberta Simiele, Marianna Caterino, Margherita Ruoppolo, Wilhelm Bloch, Robert Kraaij, André G. Uitterlinden, Alessandra Santillo, Rosalba Senese, Federica Cioffi, Elena Silvestri, Stefania Iervolino, Assunta Lombardi, Maria Moreno, Fernando Goglia, Antonia Lanni, Pieter de Lange

**Affiliations:** ^1^ Dipartimento di Scienze e Tecnologie Università degli Studi del Sannio Benevento Italy; ^2^ Dipartimento di Scienze e Tecnologie Ambientali, Biologiche e Farmaceutiche Università degli Studi della Campania Luigi Vanvitelli Caserta Italy; ^3^ Dipartimento di Medicina Molecolare e Biotecnologie Mediche Università degli Studi di Napoli Federico II Naples Italy; ^4^ Ceinge–Biotecnologie Avanzate Naples Italy; ^5^ Divulgazione Scientifica Multidisciplinare per la Sostenibilità Ricerca, Formazione Cultura (DiSciMuS RCF) Naples Italy; ^6^ Department of Molecular and Cellular Sport Medicine Institute of Cardiovascular Research and Sport Medicine German Sport University Cologne Cologne Germany; ^7^ Genetic Laboratory Department of Internal Medicine Erasmus Medical Center Rotterdam The Netherlands; ^8^ Dipartimento di Biologia Università degli Studi di Napoli "Federico II" Naples Italy

**Keywords:** exercise, food withdrawal, lipid metabolism, thermoneutrality

## Abstract

Exercise under fasting conditions induces a switch to lipid metabolism, eliciting beneficial metabolic effects. Knowledge of signaling responses underlying metabolic adjustments in such conditions may help to identify therapeutic strategies. Therefore, we studied the effect of mild exercise on rats submitted to food withdrawal at thermoneutrality (28°C) for 3 days. Animals were housed at thermoneutrality rather than the standard housing temperature (22°C) to avoid beta‐adrenergic signaling responses that themselves affect metabolism and well‐being. Quantitative analysis of multi‐organ mRNA levels, myofibers, and serum metabolites shows that this protocol (a) boosts fat oxidation in muscle and liver, (b) reduces lipogenesis and increases gluconeogenesis in liver, (c) increases serum acylcarnitines (especially C_4_OH) and ketone bodies and the use of the latter as fuel in muscle, (d) increases Type I myofibers, and (e) is associated with an increased thyroid hormone uptake and metabolism in muscle. In addition, stool microbiome DNA analysis revealed that food withdrawal dramatically alters the presence of bacterial genera associated with ketone metabolism. Taken together, this protocol induces a drastic switch toward increased lipid and ketone metabolism compared to exercise or food withdrawal alone, which may prove beneficial and may involve local thyroid hormones, which may be regarded as exercise mimetics.

## Graphical abstract

Environmental temperature influences metabolic responses. The results of the present study show that the relatively mild metabolic effects of exercise at thermoneutrality are boosted when combined with food withdrawal. To our knowledge, this is the first study to reveal this, studying the whole animal's response ranging from microbiota to muscle, liver, and serum, suggesting a contributing role for thyroid hormone.

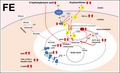

## 1 INTRODUCTION

Fasting/food withdrawal elicits rapid metabolic adaptations in skeletal muscle as well as in other organs, and this is observed both in rodents and humans (De Lange et al., 2007). Because of carbohydrate unavailability, fasting/food withdrawal causes a metabolic switch versus the use of fatty acids as fuel, with features that are reminiscent of endurance exercise (De Lange et al., 2007). Indeed, the potential beneficial effects of exercise, alone or in combination with fasting/food withdrawal on metabolic parameters, have extensively been studied both in animal models and in humans (Jaspers et al., 2017). A 6‐week protocol of exercise under fasting conditions has been shown to have beneficial metabolic effects in humans (Van Proeyen, Szlufcik, Nielens, Ramaekers, & Hespel, 1985). Rodent models are often used for in‐depth metabolic studies that are not feasible in humans, with the aim to translate the outcome to the human situation, but the influence on metabolic rates of one critical factor, environmental temperature (Fischer, Cannon, & Nedergaard, 2019), often has not been considered. Basal metabolic rates are ideally measured in the thermoneutral “comfort zone,” and data obtained from experiments in rodents performed at thermoneutrality have been argued to be most reminiscent to similar data obtained in humans (Fischer et al., 2019). However, up to now, studies on metabolic effects of fasting/food withdrawal and exercise in rodents, either including single exercise bouts (Zheng et al., 2015) or long‐term protocols based on caloric restriction/intermittent fasting and exercise (Marosi et al., 2018), are performed at temperatures inducing cold‐stress (20°C–22°C). Importantly, recent studies on mice showed that several metabolic effects of exercise are altered when animals are housed at thermoneutrality compared to room temperature (22°C), including markers of mitochondrial biogenesis and adipose browning (Aldiss et al., 2019; McKie et al., 2019), insulin sensitivity, and microbiome adaptation (Raun et al., 2019). The metabolic responses to food withdrawal at thermoneutrality were previously studied by our group in rats, both at 48 hr by transcriptional profiling (De lange et al., 2004), and over a period spanning 0 to 48 hr, with the responses resulting to be rapid and at times transient (De Lange et al., 2006). The acute metabolic response in gastrocnemius muscle involves phosphorylation of AMP‐activated protein kinase (AMPK) at Thr^172^ and increased transcription of peroxisome proliferator‐activated receptor γ coactivator‐1α(PGC‐1α), with a subsequent transcriptional increase in carnitine palmitoyl transferase 1b (CPT1b), known to induce mitochondrial fatty acid oxidation, and a delayed, sustained transcriptional response of uncoupling protein 3 (UCP3) (De Lange et al., 2006). In rodents, the metabolic effects of exercise during food withdrawal at thermoneutrality have not yet been established. We thus studied the metabolic outcome of 66 hr‐food withdrawal in male Wistar rats at thermoneutrality subjected to mild exercise (five treadmill runs for 30 min each at 15 m/min, 0° inclination). We assessed the metabolic responses of skeletal muscle and liver by determining the expression of key genes involved in energy metabolism and metabolic serum parameters including acylcarnitines, ketone bodies, and glycemia. We also assessed the effect of the above protocol on (a) serum levels of thyroid hormone, the action of which is known to partially mimic the response to exercise (Aldiss et al., 2019) and (b) compositional changes in the stool microbiome, reflecting the gut's impact on metabolism.

## 2 MATERIALS AND METHODS

### 2.1 Animal treatments, metabolic measurements, and tissue handling

Male Wistar rats (aged 3 months, weighs approximately 300 g) were housed separately at 28°C having ad libitum access to water and chow (content: fatty acids (mg/kg): palmitate (16:0) 4,387; palmitoleate (16:1) 202; stearate (18:0) 675; oleate (18:1) 5,046; linoleate (18:2) 12,335; linolenate (18:3) 1,169. Metabolizable energy (%): carbohydrates 60.4; proteins 29; fat 10.6 J/J; 15.88 KJ gross energy/g (Muscedola s.r.l., Milan, Italy). The animals were acclimated to the 28°C housing temperature for 3 weeks, being familiarized with the treadmill (Panlab, Harvard Apparatus). For each of a total of four experiments, four groups of animals were used. Each rat per group represented one separate experimental condition: the first was chow‐fed, the second was chow‐fed and submitted to exercise, the third was submitted to food withdrawal, and the fourth was submitted to food withdrawal and to exercise. All animals continued to have ad libitum access to water. Food was withdrawn for a period of 66 hr. The exercise program consisted of five low‐intensity treadmill runs (twice daily for 30 min at 15 m/min with 0° inclination, initial environmental temperature was set at 25°C and constantly monitored, temperatures inside the plexiglass cover did not exceed 28°C during the exercise sessions). After each exercise bout, the animals rested on the treadmill for 15 min and were then placed back in their cages at 28°C. Body weight and food intake were monitored during the experiment. Fresh stool from each animal was collected before the start of the experiment and on the last day, and frozen at −80°C. The duration of the experiment was 3 days, 66 hr from the start of food withdrawal to sacrifice; on the third day, the exercising animals were submitted to only one exercise bout. Subsequently, oxygen consumption (VO_2_) and carbon dioxide production (VCO_2_) were measured using a four‐chamber, indirect open‐circuit calorimeter (Columbus Instruments), with one rat per chamber at thermoneutrality (28°C) to ensure measurement of basal metabolic parameters. Measurements were performed between 1,100 and 1,400 hr. Rats were placed in separate 12.7‐L calorimetry chamber with ad libitum access to water. In order to detect metabolic rates at rest, rats were allowed to adapt to the chamber for 1 hr and the measurement of VO_2_ and VCO_2_ started when the rats were immobile for at least 10 min. The system was set at a flow rate of 1.0 L/min, a sample line‐purge time of 2 min, and a measurement period of 30 s every 12 min, for 2 hr. VO_2_ and CO_2_ values, used to calculate respiratory exchange ratio (RER), were calculated by dividing the respective rates of VCO_2_ by the corresponding rates of VO_2_ were the mean of three consecutive measurements during which rats were not moving. Thereafter, the animals were sacrificed, serum was collected, and tissues were weighed.

**Figure 1:**
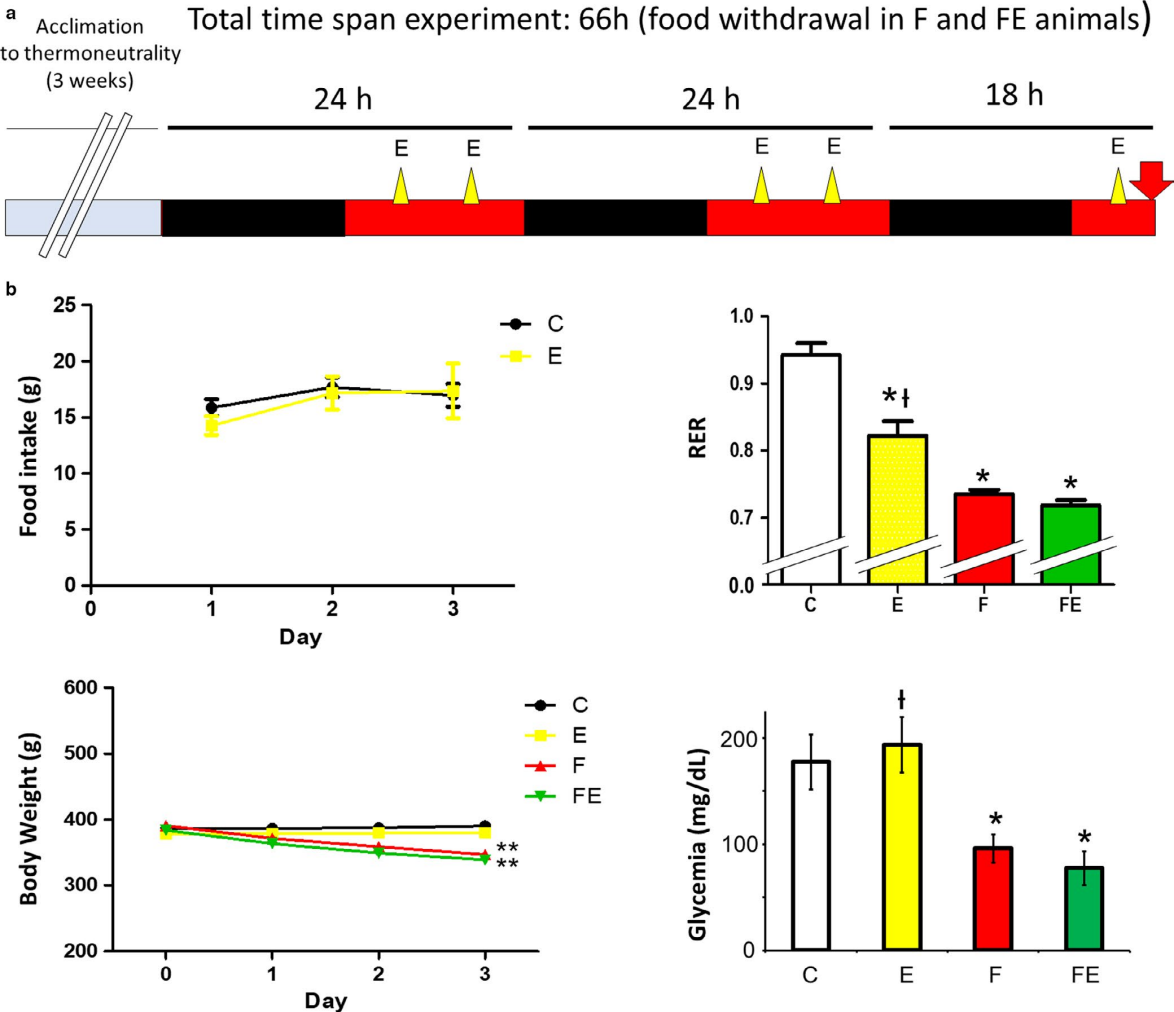
Experimental timeline (a), and food intake, body weight, metabolism, and glycemia of C, E, F, and FE rats (b). C, chow‐fed controls; E, chow‐fed exercised; F, food withdrawn; FE, food withdrawn and exercised. Black boxes indicate nocturnal periods, black and red boxes indicate food withdrawal period in F and FE rats. Yellow triangle: exercise bout. Red arrow: time of sacrifice. *p < .05 versus. C, **p < .05 versus C and E, +*p* < .05 versus F and FE. Data are shown as means ± *SEM* (each experimental condition *N* = 4)

The time span from the finish of the last exercise bout to sacrifice was 4 hr (see, for time span, Figure 1a). For immunohistochemistry, part of the gastrocnemius muscle and vastus lateralis muscle was fixed in 4% paraformaldehyde at 4°C for 24 hr, washed with 0.1 M PBS, incubated for 24 hr in 30% sucrose in 0.1 M PBS, then embedded in Tissue Tec (Varini Sr.L, Naples, Italy), frozen in liquid nitrogen, and stored at −80°C. The remaining tissue and the other tissues were immediately frozen in liquid nitrogen and stored at −80°C.

This study followed the European Commission's guidelines for laboratory animal studies, and was approved by the Ethics Committee for Animal Studies, University of Campania “Luigi Vanvitelli”, and the Ministry of Health of Italy with Project Authorization Protocol 704/2016 PR in conformance to article 31, Legislative Decree 26/2014.

### 2.2 Serum measurements

Glycemia was measured using the One Touch Verio system (Lifescan Inc). Serum‐free (f) 3,5,3′,5′‐tetraiodo‐L‐thyronine (T4) levels were measured using the ELISA kit from Diametra s.r.l., Perugia, Italy. All measurements were performed following the manufacturers’ description.

### 2.3 Serum acylcarnitine determination by liquid chromatography tandem mass chromatography (LC‐MS/MS)

Serum (10 µl) of each sample was transferred on a filter paper card and dried overnight to obtain a dried blood spot (DBS) at room temperature (Ruoppolo et al., 2018). The metabolites were extracted from DBS and esterified as previously described (Ruoppolo et al., 2015). Finally, the metabolite mixtures were resuspended in 300 μl of acetonitrile/water (70:30) containing 0.1% formic acid. The subsequent LC‐MS/MS analysis was carried out using an 1,100 series Agilent high‐performance liquid chromatograph (Agilent Technologies) and API 4000 triple quadrupole mass spectrometer (Applied Biosystems‐Sciex), by Precursor Ion Scan of 85 Da fragments. Quantitative analysis was performed by ChemoView v1.2 software (Ruoppolo et al., 2014; Scolamiero et al., 2014).

### 2.4 Serum ketone body determination by gas chromatography–mass spectrometry (GC‐MS)

Hundred microliter of each serum sample was dissolved in 500 µl of acetonitrile and centrifuged at 13,000 rpm for 30 min. Precipitated debris were discarded and supernatant was dissolved in 1.5 ml of water. The obtained solution was treated using NaOH 30% w/v to adjust pH to 14. The metabolites were oximated by adding 0.5 ml of hydroxylamine hydrochloride (2.5 mg/ml in water) at 60°C for 60 min. A small volume of 2.5 M H_2_SO_4_ was added to acidify the solution. An internal standard mix, constituted by dimethylmalonic acid (10 µM), pentadecanoic acid (10 µM), and tropic acid (10 µM), was added and then the metabolites were extracted by ethyl acetate. The organic phase was dehydrated by adding 1 g of Na_2_SO_4_ and evaporated at 40°C under a nitrogen flow. The sample was derivatized by adding N, O‐bis(trimethylsilyl) trifluoroacetamide (50 µl) at 60°C for 30 min and analyzed by GC‐MS analysis on GC column HP‐5MS; 30 m × 0.250 mm × 0.25 µm using GC‐MS Agilent 7890A (Agilent Technologies). The experimental set up was as follows: chromatographic gradient, 70°–280°C, 10°C/min; helium flow was 1 ml/min; solvent delay 6 min; scan range from 50 to 650 amu. The NIST and Wiley mass spectra library (2008), and the MSD Productivity Chemstation software (Agilent Technologies) was used to perform metabolite identification. Finally, the compound abundance was assessed comparing the areas of the chromatographic peaks versus areas of internal standard (Rossi et al., 2018).

### 2.5 Muscle fiber typing

Slides (7 µm) from gastrocnemius and vastus lateralis (LAT) samples were treated with acetone at −20°C for 5 min and incubated with 5% BSA for 30 min at room temperature and stained with primary antibody (A4951 1:250 IOWA showing Type 1 fibers) at 4°C overnight (Mathes, Vanmunster, Bloch, & Suhr, 2019). Control slides were performed in the absence of the first antibody. Thereafter, incubation with secondary antibody (goat anti mouse 1:400 DAKO) for 1 hr at room temperature was performed, followed by (after several washing steps) incubation with horseradish peroxidase (HRP) (1:400 Sigma) for 1 hr at room temperature and developed with 3,3'‐diaminobenzidine (DAB) solution until preferred staining appears. The slides were dehydrated with an alcohol lane and xylol and then covered with glass slides. The tissue sections were photographed with a light microscope (Leica Orthoplan, Leica Microsystems GmbH) using Irfanview software (Irfan Skiljan) and stored digitally as a JPG file with a resolution of 768 × 576 pixels. The image processing and analysis program "ImageJ 1.51f" (National Institutes of Health) was used to determine the fiber type distribution and calculated the ratio by quantification of stained (Type I) and unstained (Type II) fibers. In each animal, the entire area was counted covering vastus lateralis muscle and gastrocnemius muscle by at least 1.011.097 µm^2^ and 1.185.071 µm^2^, respectively. This was done in order to calculate absolute ratios between Type I and Type II fibers.

### 2.6 Gene expression analysis

cDNA was synthesized from total RNA extracted from frozen tissue, and amplification mixes were prepared as described previously (Senese et al., 2011). Real‐time PCR was carried out using the Quant Studio 5 real‐time PCR machine (Applied Biosystems, Thermo Fisher Scientific s.r.l.) using standard cycle parameters. Primers used for gene expression analysis were the following:

IGF‐1 R S: 5′‐TCCCAAGCTGTGTGTCTCTG‐3′, AS: 5′‐CTCCGTTGTTCCTGGTGTTT‐3′; IGF‐1 S: 5′‐AACCTGCAAAACATCGGAAC‐3′, AS: 5′‐GGAAATGCCCATCTCTGAAA‐3′; CS S: 5′‐CATGACGGTGGCAATGTAAG‐3′, AS: 5′‐CCATTCATAGCTGCTGCAAA‐3′; UCP3 S: 5′‐AACTGGAGGCGAGAGGAAAT‐3′, AS: 5′‐GTCCCCTGACTCCTTCTTCC‐3′; PGC‐1α S: 5′‐GTCAACAGCAAAAGCCACAA‐3′, AS: 5′‐GTGTGAGGAGGGTCATCGTT‐3′; PDK4 S:5′‐CGTCGCCAGAATTAAAGCTC‐3′, AS:5′‐TGTGGTGAAGGTGTGAAGGA‐3′; MHC I S: 5′‐GAACAGAAGCGCAATGTTGA‐3′, AS: 5′‐TCTTGCGGTCTTCCTCAGTT‐3′; MHC II S: 5′‐ATCATCCCCAATGAAACCAA‐3′, AS: 5′‐TTCTAGCACCCCGTTACACC‐3′; HK2 S: 5′‐CAGCCTAGACCAGAGCATCC‐3′, AS: 5′‐GCTTCCTTCAGCAAGGTGAC‐3′; CPT1a S: 5′‐CGGAGCAGGGATACAGAGAG‐3′, AS: 5′‐TCAAAGCATCTTCCATGCAG‐3′; CPT1b S: 5′‐ATCGAACGTGCTGCTTTCTT‐3′, AS: 5′‐ATTTGCCGTAGAGGCTGAGA‐3′; CPT2 S: 5′‐GCAATGAGGAAACCCTGAAG‐3′, AS: 5′‐GATCCTTCATCGGGAAGTCA‐3′; MCT8 S: 5′‐ACAGCGCTTTCTGGTTCAGT‐3′, AS: 5′‐AAGGCCCAGATACGGTAGGT‐3′; MCT10 S: 5′‐GTGCAATGGGTCTGTGTTTG‐3′, AS: 5′‐CCATGTTGTCATCGTCCTTG‐3′; BDNF S: 5′‐GCCCAACGAAGAAAACCATA‐3′, AS: 5′‐CAAAGGCACTTGACTGCTGA‐3′; OXCT1 S: 5′‐TGTGCAGCCATAGACTTTGC‐3′, AS: 5′‐GCACTCATGAAGCAAGACCA‐3′; ACAT1 S: 5′‐AGGTCTACATGGGCAACGTC‐3′, AS: 5′‐AGTGGCAATGGGTAGACCTG‐3′; BDH1 S: 5′‐TCTCGGACTGCCTACGCTAT‐3′, AS: 5′‐TAGAGGCTGGTGGCAGCTAT‐3′; HMGR S: 5′‐ACAAGCTGGAAGCTGGTGTT‐3′, AS: 5′‐TTAACCCATTGGAGGTGAGC‐3′; PEPCK1 S: 5′‐ACGCCATTAAGACCATCCAG‐3′, AS: 5′‐TCGATGCCTTCCCAGTAAAC‐3′; G6Pase S: 5′‐CACTCAGGAACCACCACCTT‐3′, AS: 5′‐GTGGTCAGGGAAGCAGTGAT‐3′; FAS S: 5′‐GGATGTCAACAAGCCCAAGT‐3′, AS: 5′‐CAGAGGAGAAGGCCACAAAG‐3′; ACC S: 5′‐AGCCAAGCTCAGGGACAGTA‐3′, AS: 5′‐ACGCTGAAGTAACCCCACAC‐3′; SREBP‐1c S: 5′‐GCCACCCTCTTGCTCTGTAG‐3′, AS: 5′‐GGGTGAGAGCCTTGAGACAG‐3′; DIO1 S: 5′‐AGACTGGAAGACAGGGCTGA‐3; AS: 5′‐GCCTTGAATGAAATCCCAGA‐3′; DIO2 S: 5′‐CAGTGAAGCGGAATGTCAGA‐3′, AS: 5′‐TTTCCCATTATCCCCTTTCC‐3′; B‐ACTIN S: 5′‐TGTGTTGTCCCTGTATGCCT‐3′, AS: 5′‐CCCTCATAGATGGGCACAGT‐3′.

### 2.7 Western immunoblot analysis

AMPK and phosphorylated AMPK (Thr 172) protein levels were determined in cell lysates by employing specific polyclonal antibodies (Cell Signaling Technology Inc.). The β‐actin antibody was from Bioss Antibodies Inc..

### 2.8 Stool microbiome profiling

Fresh stool samples were collected on days 0 and 3 and stored at −80°C. One stool excrement was weighed (~100 mg) and DNA was isolated from the samples using the InviMag Stool DNA Kit (Invitek Molecular) on the KingFisher robot (Thermo Fisher Scientific) after bead beating using 0.1‐mm silica beads (MP Biomedicals, LLC, Bio Connect Life Sciences BV) with four large zirconia beads II added to the tube for homogenization (Invitek Molecular). The V3 and V4 hypervariable regions of the 16s rRNA gene were amplified using the 308F‐806R primer pair and dual indexing as described in (Fadrosh et al., 2014). Amplicons were normalized, purified (Agencourt AMPure XP, Beckman Coulter Life Science), and pooled prior to sequencing. Size and quantity of the pooled amplicon were assessed (LabChip GX, PerkinElmer Inc.), and a control library was added at ~10% of the amplicon pool (PhiX Control v3 library, Illumina Inc.) followed by paired‐end (2 × 300 bp) sequencing on the MiSeq platform (Illumina Inc.) with an average depth of 50,000 paired reads per sample. Samples were sequenced in two separate sequencing runs. Data processing, quality control, and taxonomic classification were done using an in‐house pipeline (µRAPtor) based on QIIME version 1.9.045 (Caporaso et al., 2010) and UPARSE version 8.146 (Edgar, 2013). Reads were merged and low‐quality and chimeric reads were excluded. A direct classification of the merged reads using RDP classifier [(2.12; Wang, Garrity, Tiedje, & Cole, 2007)] and the SILVA 16S database [(release.128; Quast et al., 2012)] was done to reconstruct taxonomic composition. Samples were normalized to 10,000 random reads, singleton taxa were excluded, and Shannon alpha diversities were calculated (QIIME) followed by more stringent taxa filtering of all taxa with <10 reads. This resulted in a table with 535 taxa. For each higher taxonomic level (domain, phylum, class, order, family, and genus), a table was generated by binning (QIIME).

### 2.9 Statistical analysis

Sample size was calculated using the G*Power software version 3.1.9.2 from the Heinrich Heine University of Dusseldorf (http://www.gpower.hhu.de). The results from the power test were that in order to compare four groups and observe expected effects within the physiological range, the number of animals to be used in each group is four. The chosen α was 0.5 and the power was 0.90, which resulted in an effect size of 1.27, implying that four different samples from each group are sufficient to observe statistically significant differences with the parameters set as such using one‐way ANOVA (post hoc test: Student–Newman–Keuls). Rats undergoing exercise sessions (control exercise and food withdrawal exercise groups) had to satisfy the following inclusion criteria: all the animals had to complete five treadmill runs without interruptions. Thus, animals that refused to run or pause at irregular time intervals (this occurs frequently with forced short‐term exercise experiments using treadmills) were excluded from the analysis. Six rats were exercised per condition (with or without food withdrawal, respectively) and four of them completed all five treadmill runs without interruptions. Analyses on microbiome data were performed in R (R Core Team, 2017) using R package vegan (Oksanen et al., 2013). Changes in alpha diversity were calculated per rat by subtracting the day 3 value by the day 0 value. Differences between the groups were assessed by *t* test. Beta diversities (Bray–Curtis dissimilarity) were calculated in vegan, graphed as principal coordinate ordination, and the effect sizes and corresponding significance of the different groups on this ordination were determined using PERMANOVA (adonis function in vegan) while controlling for sequencing read depth and sequencing run covariates of the samples. Compositional changes at family and genus levels were calculated per taxon by subtracting the day 3 abundance by day 0 abundance per rat. Correlation of changes per taxon with the four groups was assessed by linear regression (lm function in R) without controlling, and significant taxa were clustered based on the pair‐wise Euclidean distances and presented as a heatmap (heatmap.2 function in R). Differences were considered significant at *p* < .05.

All other data (serum parameters, gene expression data, fiber typing, and Western blotting data) are expressed as means ± *SEM*. Statistical differences of normally distributed data between two treatments were determined by unpaired Student's *t* test, or for more treatments by one‐way ANOVA (post hoc test: Student–Newman–Keuls), using Prism 5.0 (Graphpad). Differences between treatments were considered significant at *p* < .05.

## 3 RESULTS

### 3.1 Exercise does not change food intake and body weight, conserves muscle weight, maintains the food withdrawal‐induced complete shift toward fat use, and does not alter the drop in glycemia caused by food withdrawal

Exercise did not increase food intake over time (Figure 1b), neither did it reduce body weight (Figure 1b). With respect to chow‐fed control RERs (0.94 ± 0.02, which indicates almost exclusive carbohydrate use), RERs induced by exercise were lowered by 13%, indicating a partial shift toward fat use, and by 21% upon food withdrawal, indicating a complete switch toward fat use, which did not significantly change with food withdrawal/exercise (lowering of 23% with respect to chow‐fed controls, Figure 1b). Glycemia (mg/dl) did not change with exercise, and the 1.9‐fold drop in glycemia upon food withdrawal changed to 2.3‐fold with food withdrawal/exercise, without reaching statistical significance versus food withdrawal (Figure 1b). Both food withdrawal and food withdrawal/exercise reduced liver weight by 40% (Table 1) and both visceral and subcutaneous white adipose tissue weights (g) were equally reduced by exercise, food withdrawal, and food withdrawal/exercise (by 22 and 44%, respectively, Table 1), and muscle weight was neither significantly affected by exercise, food withdrawal, nor by food withdrawal/exercise (Table 1).

**Table 1:** Organ weights of the different treatment groups

WEIGHT (g)	C	E	F	FE
Liver	10.66 ± 0.51	9.7 ± 0.49	6.43 ± 0.25**	6.61 ± 0.29**
Heart	0.93 ± 0.03	0.98 ± 0.04	0.8 ± 0.03	0.89 ± 0.03
Gastrocnemius	2.05 ± 0.11	2.1 ± 0.12	1.92 ± 0.05	1.8 ± 0.06
Soleus	0.13 ± 0.01	0.16 ± 0.02	0.12 ± 0.01	0.15 ± 0.01
EDL	0.16 ± 0.002	0.14 ± 0.001	0.16 ± 0.001	0.15 ± 0.01
LAT	0.99 ± 0.1	1.14 ± 0.17	1.15 ± 0.15	1.11 ± 0.04
WAT visc	2.28 ± 0.45	1.8 ± 0.4*	1.77 ± 0.39*	1.79 ± 0.19*
WAT subc	6.02 ± 0.49	3.69 ± 0.69*	3.35 ± 0.49*	3.5 ± 0.48*
BAT	0.45 ± 0.04	0.35 ± 0.04	0.26 ± 0.03	0.38 ± 0.07

Note

Data are shown as means ± *SEM* (each experimental condition *N* = 4).

Abbreviations: BAT, brown adipose tissue (intrascapular depot); C, chow‐fed controls; E, chow‐fed exercised; EDL, extensor digitorum longus; F, food withdrawn; FE, food withdrawn and exercised; WAT visc, total visceral white adipose tissues (mesenteric, gonadic, perirenal, retroperitoneal); WAT subc, subcutaneous white adipose tissue (flank adipose tissue depot).

* *p* < .05 versus C,

** *p* < .05 versus E and C.

### 3.2 The combination of food withdrawal and exercise increases Type I/Type II myofiber ratio, modulates transcription toward increased use of fat and ketone bodies, and increases AMPK Thr^172^ phosphorylation in gastrocnemius muscle

**Figure 2:**
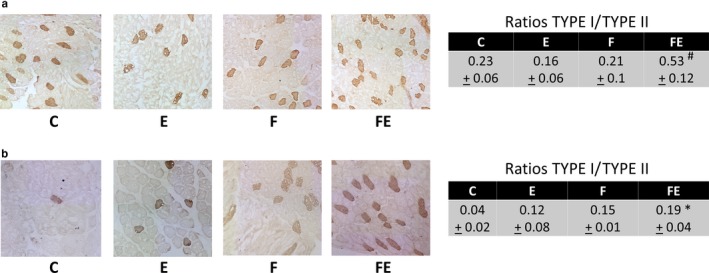
Representative fields of immunohistochemical stained portions of gastrocnemius (a) and vastus lateralis (b) muscle for Type I myofiber abundance in rats from the different experimental conditions indicated below the images. C, chow‐fed controls; E, chow‐fed exercised; F, food withdrawn; FE, food withdrawn and exercised. *p < .05 versus. C, #*p* < .05 versus. C and E. Data are shown as means ± *SEM* (each experimental condition *N* = 4)

**Figure 3:**
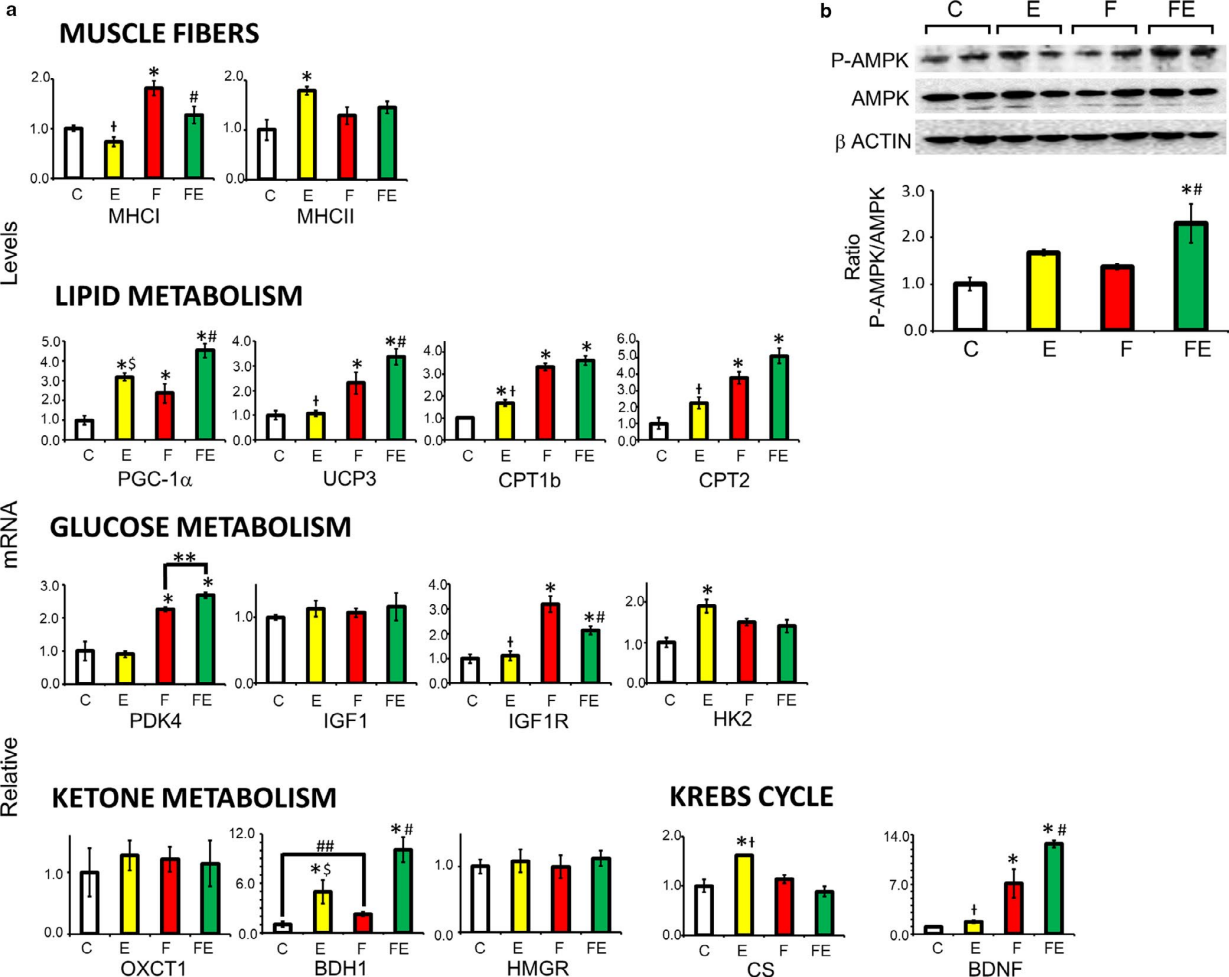
Expression of genes involved in lipid, glucose, and ketone metabolism, and BDNF in gastrocnemius muscle, and AMPK phosphorylation 4 hr after the last exercise bout (E and FE). (a): Gene expression levels, (b): Western analysis of AMPK phosphorylation at Thr^172^. A representative membrane showing two samples for each condition is shown. The histogram underneath represents the mean P‐AMPK/AMPK ratio obtained from each rat. C, chow‐fed controls; E, chow‐fed exercised; F, food withdrawn; FE, food withdrawn and exercised. *p < .05 versus C, $*p* < .05 versus FE, †*p* < .05 versus F, FE, #*p* < .05 versus F., [**p = .048 vs. F, ##*p* = .044 vs. C (Student's unpaired *t* test)]. Data are shown as means ± *SEM* (each experimental condition *N* = 4)

In response to food withdrawal/exercise, Type I myofiber abundance versus chow‐fed controls was increased in both gastrocnemius (Type I/Type II ratios increasing 2.3‐fold, Figure 2a) and vastus lateralis (Type I/Type II ratios increasing 4.8‐fold, Figure 2b). In gastrocnemius muscle, an additional increase was observed with respect to exercise alone (Type I/Type II ratios increasing 3.3‐fold, Figure 2b). Since Type I/fiber ratios were 3‐fold higher upon food withdrawal/exercise in gastrocnemius muscle with respect to vastus lateralis muscle, we chose to continue using gastrocnemius muscle for further analysis. As shown in Figure 3a, in analogy to the above, exercise reduced gastrocnemius muscle transcription of myosin heavy chain I (MHCI) with respect to food withdrawal by 1.2‐fold. In turn, food withdrawal increased MHCI transcription 1.8‐fold versus chow‐fed controls and 1.4‐fold versus food withdrawal/exercise. Instead, exercise increased myosin heavy chain II (MHCII) transcription 1.8‐fold versus chow‐fed controls, whereas no difference was observed in food withdrawal and food withdrawal/exercise. We next studied mRNA levels of genes involved in lipid metabolism (Figure 3a). With respect to chow‐fed controls, exercise induced PGC‐1α transcription more (3.2‐fold) compared to food withdrawal (2.4‐fold), food withdrawal/exercise showing an additive increase (4.5‐fold). Exercise did not increase UCP3 transcription, which was increased by 2.3‐fold by food withdrawal and by 3.4‐fold by food withdrawal/exercise (Figure 3a). With respect to chow‐fed controls, exercise increased CPT1b expression 1.7‐fold, food withdrawal 3.3‐fold, and food withdrawal/exercise 3.6‐fold, and exercise increased carnitine palmitoyl transferase 2 (CPT2) expression 2.2‐fold, food withdrawal 3.8‐fold, and food withdrawal/exercise 5.1‐fold. Regarding glucose metabolism, pyruvate dehydrogenase kinase 4 (PDK4) transcription was not induced by exercise but was increased 2.3‐fold by food withdrawal, and this value increased with exercise/food withdrawal with respect to food withdrawal alone (increase with respect to chow‐fed controls: 2.7‐fold). Unlike that insulin‐like growth factor (IGF1), the expression of its receptor, IGF1R, was upregulated both by food withdrawal (3.2‐fold) and food withdrawal/exercise (2.1‐fold). Hexokinase 2 (HK2) transcription was only increased by exercise (1.9‐fold). Of the genes involved in ketone metabolism, 3‐hydroxybutyrate dehydrogenase 1 (BDH1), inducing ketolysis, was increased 5.0‐fold by exercise, 2.2‐fold by food withdrawal, but a 10.1‐fold more by food withdrawal/exercise, indicating muscle increasingly uses ketone bodies as fuel in this condition. Transcription of citrate synthase (CS), the rate limiting enzyme of the Krebs cycle, was only increased by exercise (1.6‐fold). Interestingly, brain‐derived neurotrophic factor (BDNF) expression was induced 7.1‐fold by food withdrawal and this effect was drastically increased to 12.7‐fold with food withdrawal/exercise. Food withdrawal/exercise induced an increase in AMPK phosphorylation at Thr^172^ versus chow‐fed controls (2.3‐fold) and food withdrawal alone (1.7‐fold) but not versus exercise, which itself failed to significantly induce AMPK phosphorylation (Figure 3b).

### 3.3 The combination of food withdrawal and exercise modulates transcription toward increased fat oxidation, decreased lipogenesis, and increased gluconeogenesis in live

**Figure 4:**
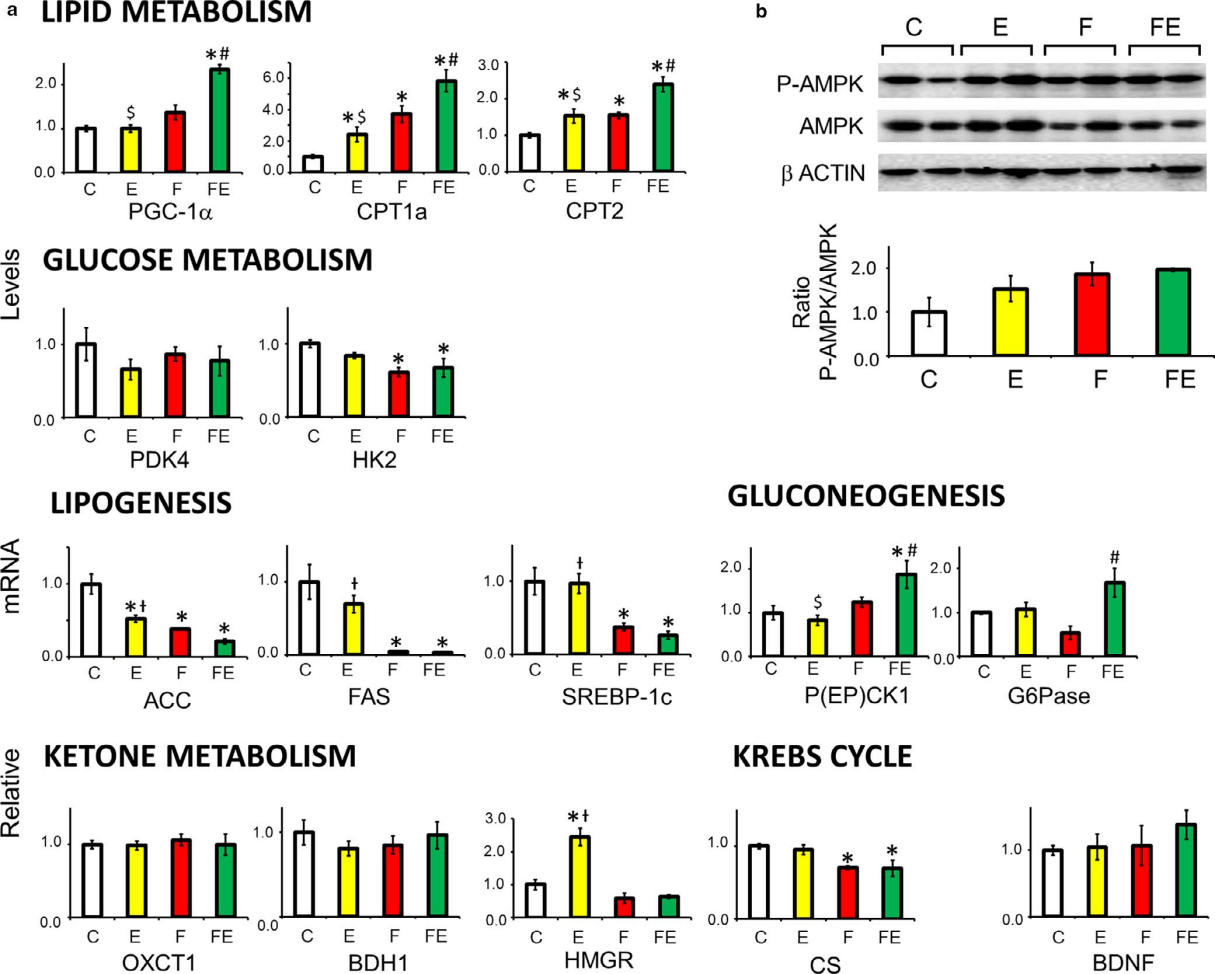
Expression of genes involved in lipid, glucose, and ketone metabolism, BDNF, and AMPK phosphorylation in liver 4 hr after the last exercise bout (E and FE). (a): Gene expression levels, B: Western analysis of AMPK phosphorylation at Thr^172^. A representative membrane showing two samples for each condition is shown. The histogram underneath represents the mean P‐AMPK/AMPK ratio obtained from each rat. C, chow‐fed controls; E, chow‐fed exercised; F, food withdrawn; FE, food withdrawn and exercised. *p < .05 versus C, $*p* < .05 versus FE, †*p* < .05 versus F; FE, #*p* < .05 versus F. Data are shown as means ± *SEM* (each experimental condition *N* = 4)

As shown in Figure 4a, with respect to controls, liver transcription of PGC‐1a was 2.3‐fold increased by food withdrawal/exercise, and so were those for CPT1a and CPT2 (by 5.8‐ and 2.4‐fold, respectively). Transcription of the latter two genes was induced to a lesser extent by exercise (2.4‐ and 1.5‐fold, respectively) and food withdrawal alone (3.7‐ and 1.6‐fold, respectively), which implies that the combined stimuli caused an additive effect. HK2 expression was decreased both by food withdrawal (1.7‐fold) and exercise/food withdrawal (1.4‐fold). With respect to chow‐fed controls, expression of genes involved in lipogenesis acetyl‐CoA carboxylase (ACC), fatty acid synthase (FAS), and sterol regulatory element‐binding protein‐1c (SREBP‐1c) was increasingly reduced by exercise (1.9‐fold, 1.44‐fold, unchanged, respectively), food withdrawal (2.9‐fold, 22.0‐fold, 2.7‐fold, respectively), and food withdrawal/exercise (4.7‐fold, 29.0‐fold, 3.8‐fold, respectively). Food withdrawal/exercise increased the expression of liver gluconeogenic genes phosphoenolpyruvate carboxykinase (PEPCK1) (1.9‐fold) and glucose‐6‐phosphatase (G6Pase) (1.7‐fold). CS transcription was lowered both by food withdrawal and by food withdrawal/exercise (both 1.4‐fold). With regard to ketone metabolism, only exercise increased the transcription of HMG‐CoA reductase (HMGR) (2.4‐fold). Transcription of liver succinyl‐CoA:3‐ketoacid coenzyme A transferase 1 (OXCT1) and BDH1 did not vary under each condition. This was also true for BDNF, which was expressed at very low levels in liver. Although AMPK phosphorylation tended to increase by food withdrawal/exercise in liver with respect to chow‐fed controls, this difference did not reach statistical significance (Figure 4b).

### 3.4 Serum levels of acylcarnitines and ketone bodies indicate that the combination of exercise and food withdrawal at thermoneutrality strongly increases incomplete fatty acid oxidation

**Figure 5:**
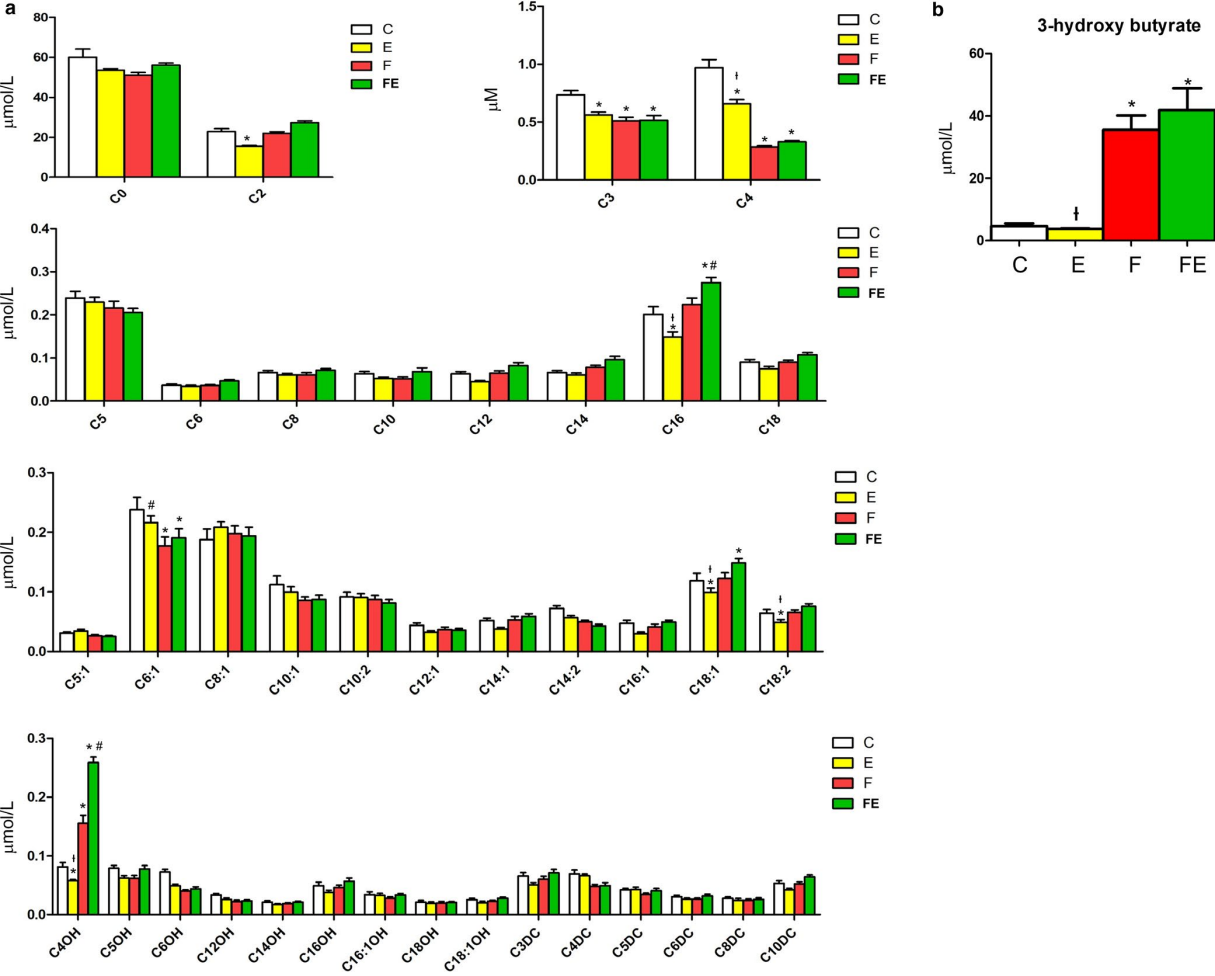
Acylcarnitines and 3‐hydroxy butyrate accumulating in the serum of C, E, F, and FE rats. C, chow‐fed controls; E, chow‐fed exercised; F, food withdrawn; FE, food withdrawn and exercised. *p < .05 versus C, $*p* < .05 versus FE, †*p* < .05 versus F, FE, ^#^ *p* < .05 versus F. Data are shown as means ± *SEM* (each experimental condition *N* = 4)

With respect to chow‐fed controls, both serum palmitoyl carnitine (C16) and oleoyl carnitine (C18:1) acylcarnitine levels were decreased by exercise (1.4‐fold and 1.2‐fold, respectively), unaltered by food withdrawal, and increased by exercise and food withdrawal (1.4‐fold and 1.2‐fold, respectively) (Figure 5a). This indicates that exercise boosts the food withdrawal‐induced use of fat as fuel. Incomplete fatty acid oxidation increased in food withdrawal/exercise versus exercise (which by itself induced a more complete oxidation with respect to chow‐fed controls) and food withdrawal alone. Of note, with respect to chow‐fed controls, serum levels of 3‐hydroxybutyrylcarnitine (C_4_OH) significantly decreased 1.4‐fold with exercise, increased 2‐fold with food deprivation, and 3.3‐fold with food withdrawal/exercise. Considering the increased level of C_4_OH, known as a marker for 3‐hydroxybutyrate (Hack et al., 2006), complementary analysis was performed to quantify 3‐hydroxybutyrate levels, which were increased 8‐fold by food withdrawal with respect to chow‐fed controls, and this value did not significantly change with food withdrawal/exercise (Figure 5b).

### 3.5 Decreased serum fT4 levels upon food withdrawal are normalized by food withdrawal/exercise, and transport/activation of thyroid hormone is regulated in a tissue‐specific manner

**Figure 6:**
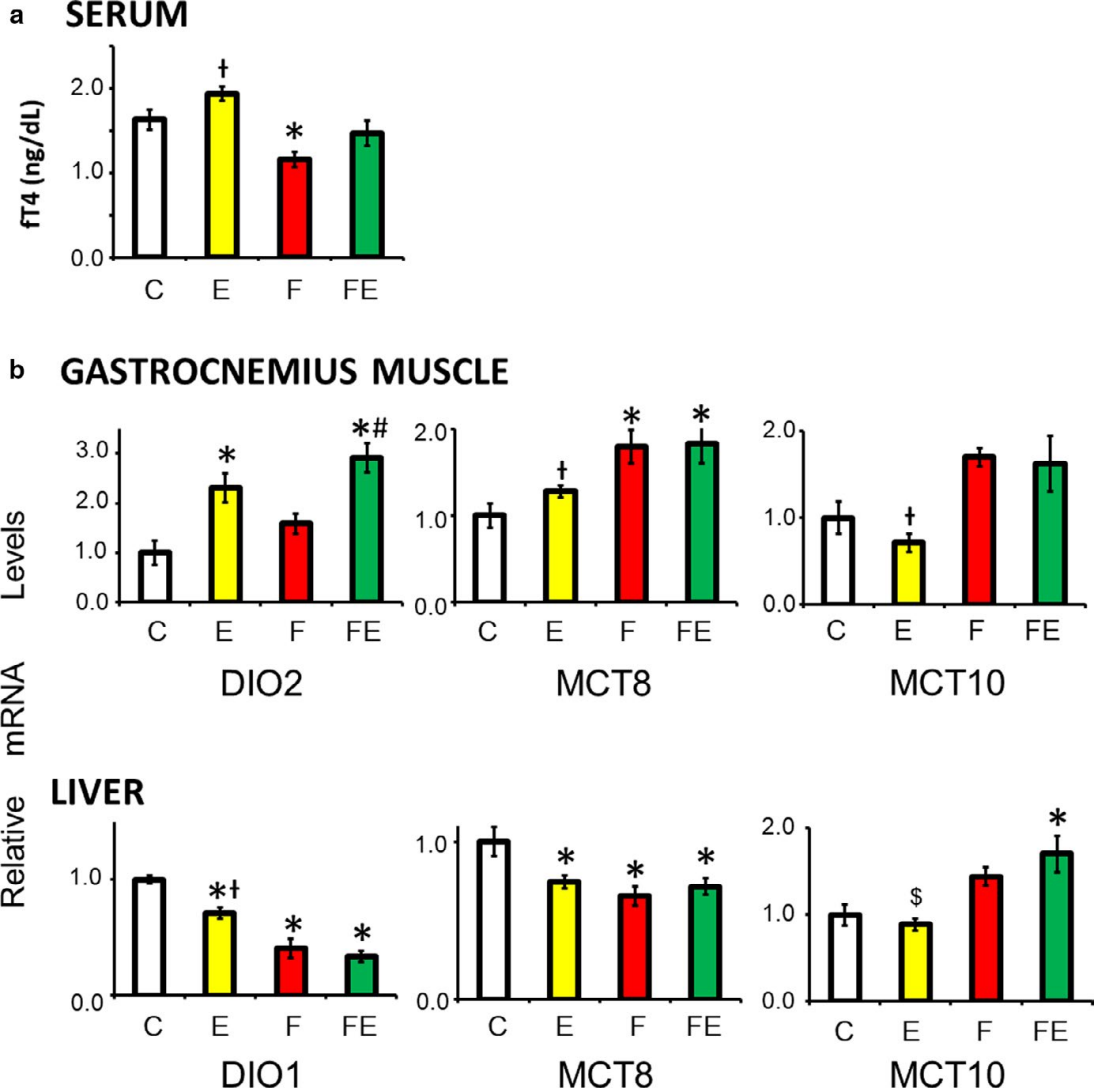
Effect of exercise, food withdrawal, and food withdrawal and exercise on serum thyroid hormone levels, and thyroid hormone transport and metabolism‐related expression in gastrocnemius muscle and liver, 4 hr after the last exercise bout (E and FE). Analyses of serum thyroid hormone levels (a), and transcription of genes involved in thyroid hormone uptake and conversion in gastrocnemius muscle and liver (b) are shown. C, chow‐fed controls; E, chow‐fed exercised; F, food withdrawn; FE, food withdrawn and exercised. *p < .05 versus C, +*p* < .05 versus F, FE, $*p* < .05 versus FE, #*p* < .05 versus F. Data are shown as means ± *SEM* (each experimental condition *N* = 4)

With respect to chow‐fed controls, only food withdrawal decreased serum‐free fT4 levels (1.4‐fold, Figure 6a). Figure 6b shows that gastrocnemius muscle transcription of DIO2, converting T4 into 3,5,3′‐triiodo‐L‐thyronine (T3), was upregulated 2.3‐fold by exercise and 2.9‐fold more by food withdrawal/exercise. Food withdrawal and food withdrawal/exercise increased the transcription of the thyroid hormone transporter, monocarboxylate transporter 8 (MCT8), with respect to chow‐fed controls (both 1.8‐fold) and exercise (both 1.4 ‐fold). Transcription of MCT10 was also increased by food withdrawal and food withdrawal/exercise with respect to exercise (2.4‐fold and 2.3‐fold, respectively). In liver, instead, exercise reduced the expression of deiodinase 1 (DIO1), and even more so did food withdrawal and food withdrawal/exercise (1.4‐fold, 2.4‐fold, and 3.0‐fold, respectively). With respect to chow‐fed controls, transcription of liver MCT8 was decreased in all conditions (1.4‐fold, 1.5‐fold, and 1.4‐fold, respectively) but monocarboxylate transporter 10 (MCT10) transcription was increased by food/withdrawal/exercise (1.7‐fold) (Figure 6b).

### 3.6 Food withdrawal induces drastic beneficial variations in stool microbiome composition, which are maintained by food withdrawal/exercise

**Figure 7:**
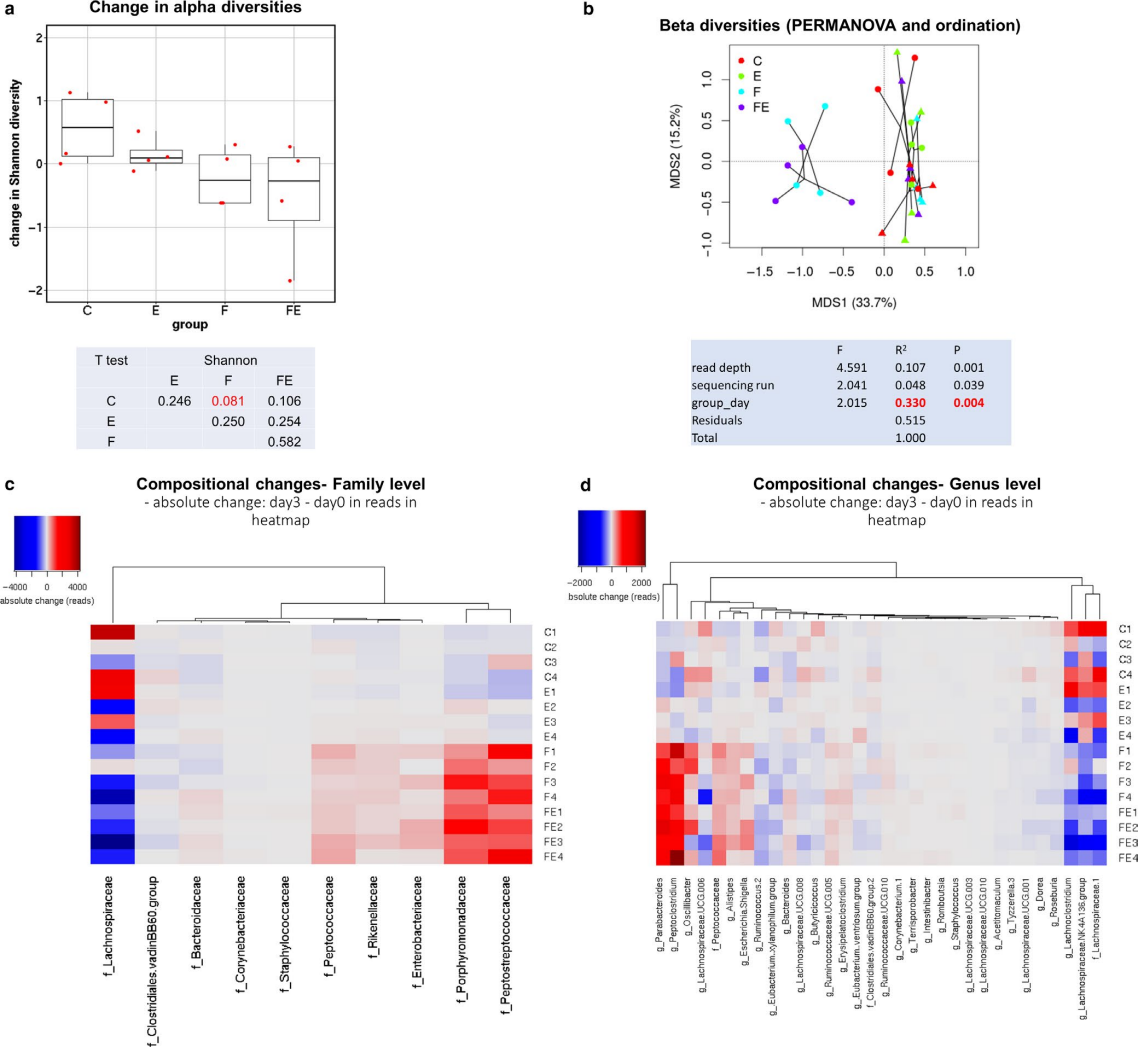
Effect of food withdrawal and exercise (FE) on stool microbiome composition. (a): Change in alpha diversity (day 3 ‐ day 0) for the four groups (each experimental condition *N* = 4). Individual Shannon alpha diversity values are represented by red points; the median is presented in the box. T test p values between the groups are presented in the table. (b): Sample clustering based on Bray–Curtis dissimilarities is represented in an ordination plot. Samples are colored per group and connected per time point; triangles represent day 0 samples, circles represent day 3 samples. PERMANOVA model to determine the effect of the groups on the overall compositions based on Bray–Curtis dissimilarities. (c): Absolute compositional changes (day 3–day 0) in reads at family level. Only families that were significantly different between the groups are presented. D: As C, at genus level. C, chow‐fed controls; E, chow‐fed exercised; F, food withdrawn; FE, food withdrawn and exercised

Analysis of the microbiome composition changes (day 3–day 0) of the stool from the four experimental groups revealed that individual Shannon alpha diversity values did not vary significantly, although a trend of decreasing alpha diversity was observed in the food‐withdrawn and exercised/food withdrawn groups (Figure 7a). The Bray–Curtis dissimilarity ordination plot shows that conditions involving food withdrawal induce a clearly distinguishable compositional change that explained 33% of the variation in overall stool microbiome composition (PERMANOVA *p* < .004) with respect to the other conditions (Figure 7b). At the family level, an increase of over 1000‐fold in Porphyromonadaceae and Peptostreptococcaceae and a decrease in Lachnospiraceae were identified (Figure 7c). Especially genera Peptoclostridium and Parabacteroides were drastically upregulated and several genera from the Lachnospiraceae family were downregulated over 1000‐fold (Figure 7d) for both groups that included food withdrawal in their regimen. The same results were obtained when relative abundances were assessed for day 3 only (Figure S1), with the addition of the less drastically downregulated genus Lachnoclostridium.

## 4 DISCUSSION

We show here that food withdrawal at thermoneutrality modulates the response of mild exercise toward a strong oxidative stimulus. Metabolic data showed exercise alone only partially shifted fuel use versus fat, whereas in combination with food withdrawal, the metabolic switch versus fat was complete. In analogy, the transcriptional response toward a switch versus fatty acid oxidation by mild exercise and by food withdrawal at thermoneutrality was enhanced when both stimuli were combined (e.g., PGC‐1α in muscle and liver, and PDK4 and UCP3 in muscle), which indicates that long‐term responses are programed, with a lasting effect that prevails after the initial stimuli cease. This is underlined by our findings that food withdrawal/exercise significantly increased muscle Type I/Type II fiber ratios and induced muscle AMPK phosphorylation. As expected, no increase in muscle AMPK phosphorylation was observed after 66 hr of food withdrawal alone, since under these conditions AMPK phosphorylation is transient, both in rats at thermoneutrality (De Lange et al., 2006) and mice at 22°C (Brockhoff et al., 2017). We have previously shown that ablation of UCP3 is associated with reduced fatty acid oxidation in mice, both at 24°C (Senese et al., 2011) and at thermoneutrality (30°C for mice) (Lombardi et al., 2019). In analogy, training in humans during fasting increased muscular oxidative capacity more than during carbohydrate supply (Van Proeyen et al., 1985), associated with increased phosphorylation of AMPK and increased UCP3 transcription (De Bock et al., 2005).

**Figure 8:**
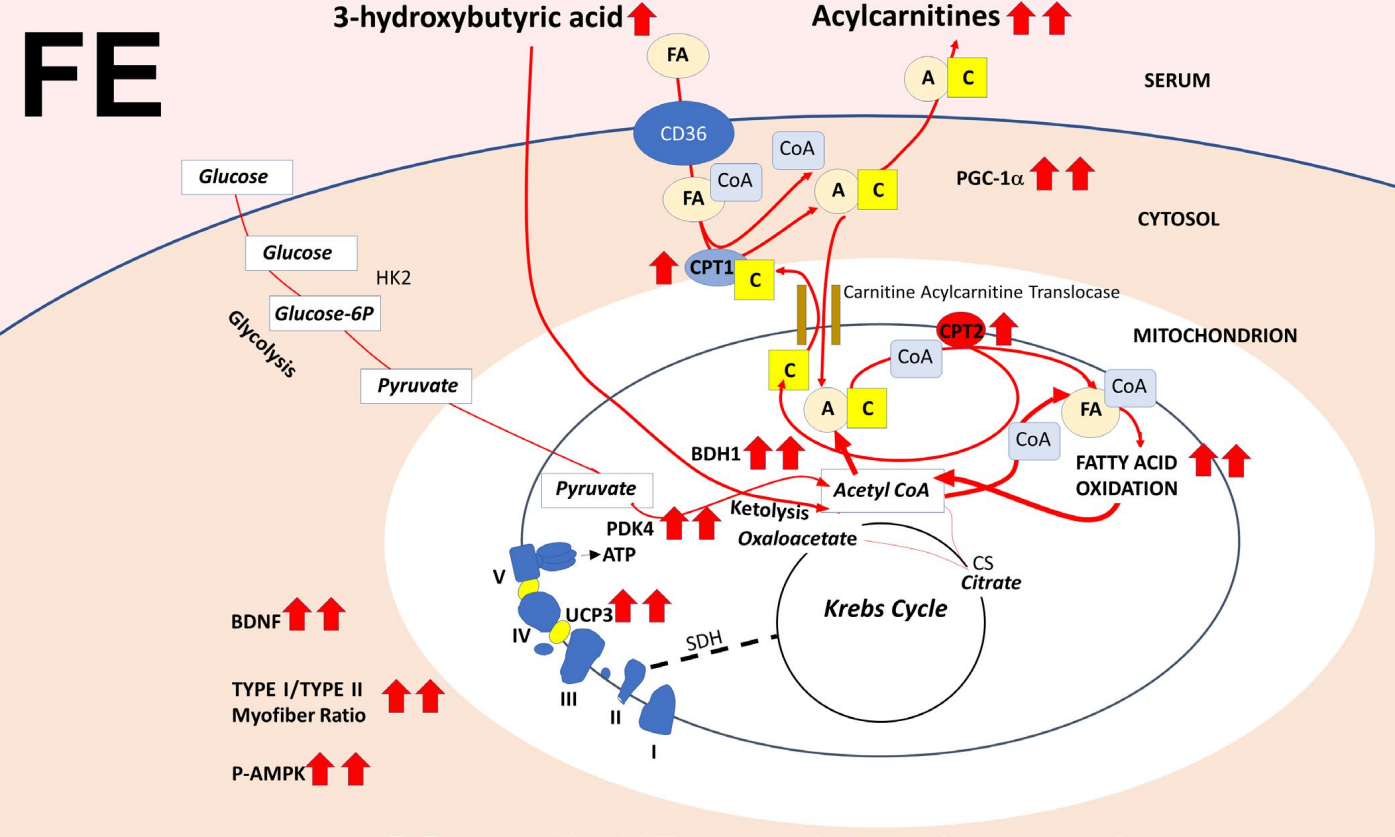
Combined effect of food withdrawal and exercise (FE) on transcription of genes involved in fuel metabolism in rat gastrocnemius muscle. Arrows depict increased transcription of genes, release or uptake of metabolites in/from the serum, Type I versus Type II myofiber ratio, and AMPK phosphorylation. Double arrows underline the additive effect of exercise. For abbreviations: see text. SDH: succinate dehydrogenase

**Figure 9:**
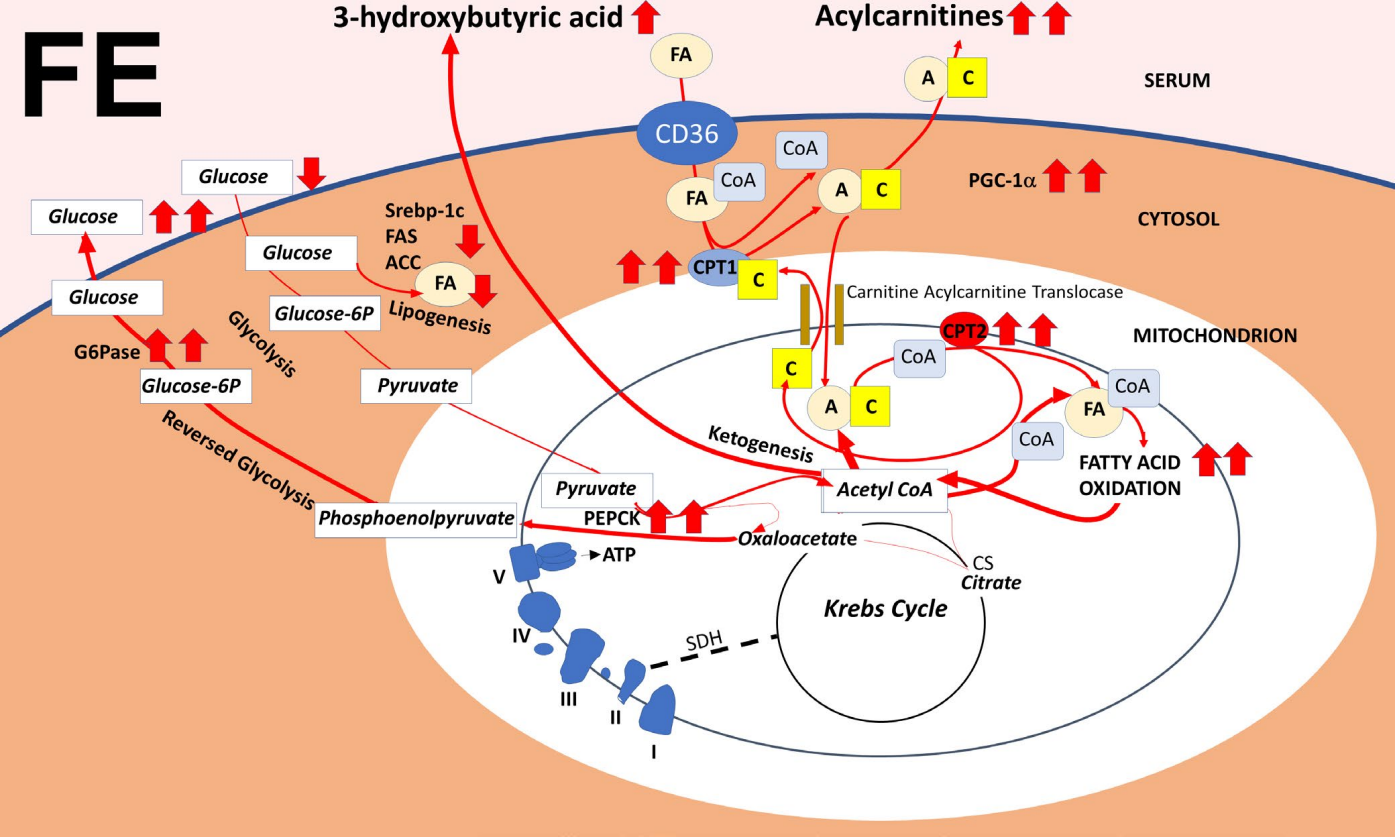
Combined effect of food withdrawal and exercise (FE) on transcription of genes involved in fuel metabolism in rat liver. Upward arrows and downward arrows depict increased or decreased transcription of genes, and release of metabolites in the serum, respectively. Double arrows underline the additive effect of exercise. For abbreviations: see text. SDH, succinate dehydrogenase

Although exercise did increase transcription of muscle CS, no increase was observed when exercise was combined with food withdrawal. In liver, CS transcription was reduced by food withdrawal and food withdrawal/exercise. As confirmed by serum parameters, an important outcome of this study is that this mild exercise stimulus, although by itself increasing efficiency of fatty acid oxidation, in combination with food withdrawal proved to be sufficient to induce an overflow of fatty acid oxidation over their further processing via the Krebs cycle, exceeding that caused by food withdrawal alone. Acylcarnitines, like ketones, are the result of incomplete fatty acid oxidation, and accumulate and modulate insulin sensitivity in the muscle cell upon fatty acid overload (Aguer et al., 2015; Giacco et al., 2019). As expected from the above, exercise induced a reduction of the serum acylcarnitines C16 and C18:1 with respect to chow feeding and food withdrawal, and the combination of both stimuli increased their levels. One acylcarnitine, C_4_OH, is a known marker for 3‐hydroxy butyrate levels (Hack et al., 2006). Despite the sharp increase in serum C_4_OH levels with food withdrawal/exercise over that seen with food withdrawal alone, serum 3‐ hydroxy butyrate levels were maximal upon food withdrawal and did not change with food withdrawal/exercise. This can be explained by the fact that ketone bodies are a fuel source when carbohydrates are low, in brain as well as in skeletal muscle. Indeed, expression of gastrocnemius muscle ketolytic gene BDH1 sharply increased upon food withdrawal/exercise, indicating an additional fuel switch toward ketone usage in muscle with respect to food withdrawal alone, in which BDH1 increased to a much lesser extent. It has been shown that PGC1‐α directly controls the expression of the ketolytic genes in skeletal muscle (Svensson, Albert, Cardel, Salatino, & Handschin, 2016). This in agreement with the additive effect of exercise and food withdrawal on increased PGC1‐α and BDH1 expression (see, for an overview: Figure 8). An alternative way to increase ketone usage is consumption of ketogenic diets. Of note, although effectively increasing serum ketone bodies without inducing obesity, ketogenic diets have been shown to result in hepatic steatosis in animal models, with controversial results in humans (Kosinski & Jornayvaz, 2017). Instead, it is well known that exercise counteracts nonalcoholic fatty liver disease (NAFLD) (Haczeyni et al., 2015). Indeed, our data reveal that exercise stimulated the reduction of transcription of hepatic lipogenic genes ACC, FAS, and SREBP‐1c by food withdrawal, thus exercise and food withdrawal combine increased ketone body metabolism with reduced fat accumulation in liver. Finally, a further drop in food withdrawal‐induced glycemia was prevented by the exercise‐induced increased transcription of hepatic PEPCK and G6Pase, both encoding key enzymes in the synthesis and release of glucose from oxaloacetate (see, for an overview, Figure 9). The lack of increase in liver AMPK phosphorylation at the measured time point in the animals subjected to exercise and food withdrawal indicates that ATP depletion is more prominent in the working muscle than in the liver under these conditions. BDNF in exercised muscle has been associated with muscle regeneration (Yu, Chang, Gao, Li, & Zhao, 2017), and with an increased oxidative profile, inducing AMPK phosphorylation (Pedersen et al., 2009), features that we found to be blunted at thermoneutrality but to be enhanced by food withdrawal at thermoneutrality, since we observed a sharp increase in the expression of muscle BDNF by food withdrawal/exercise in association with AMPK phosphorylation. Increased BDNF expression during food withdrawal and food withdrawal/exercise correlates with the elevated serum levels of 3‐hydroxy butyrate, which has been shown to induce the expression of this gene by activating its promoter regions in muscle (Sleiman et al., 2016).

We previously found UCP3 to be a transcriptional target of T3 in skeletal muscle and heart, and to be associated with increased resting metabolic rate and mitochondrial fatty acid oxidation at thermoneutrality induced by T3 (De Lange et al., 2001). In analogy to what we show here to occur with exercise, we previously showed that T3 at thermoneutrality further induces muscle UCP3 transcription during food withdrawal (Moreno et al., 2003). Food withdrawal/exercise normalized serum fT4 levels that were reduced by food withdrawal. We measured fT4 levels since these most closely represent the quantity of readily available thyroid hormone to be taken up into the cells. fT3 levels would represent both thyroid‐derived T3 and T3 converted from T4 taken up by the peripheral tissues. Interestingly, local synthesis of T3 from T4 in muscle by increased deiodinase 2 (DIO2) activity has been shown to be essential for the response to exercise through PGC‐1α (Bocco et al., 2016). The fact that food withdrawal/exercise boosted muscle PGC‐1α transcription, and the observations that (a) food withdrawal increased the expression of both thyroid hormone transporters MCT8 and MCT10 in gastrocnemius muscle and that this was maintained in response to exercise, and (b) exercise increased transcription of DIO2, and more so in combination with food withdrawal suggest that local T3 plays a role in the muscle's response to exercise during food withdrawal. Interestingly, despite the increased Type I/Type II fiber ratio seen with food withdrawal/exercise, the increased MHCI transcription induced by food withdrawal was normalized by exercise. This apparent paradox may be due to a direct effect of the increased local T3 levels on MHCI transcription, in analogy to what has been observed in the heart (Edwards, Bahl, Flink, Cheng, & Morkin, 1994); however, we have previously shown that gastrocnemius muscle MHCI protein levels diminish in response to T3 after at least 12 hr at thermoneutrality in the rat (Lange et al., 2008), a time span which exceeds that of the response to exercise assessed here (4 hr). Instead, DIO1 expression was reduced in liver in response to exercise, food withdrawal, and its combination. Food withdrawal has been previously shown to reduce MCT8 and MCT10 expression in mouse liver at 22°C (Schutkowski, Wege, Stangl, & König, 2014). In our study performed at thermoneutrality, food withdrawal (as did exercise and food withdrawal/exercise) decreased the expression of hepatic MCT8; however, food withdrawal did not change MCT10 expression, and it increased with food withdrawal/exercise. Thus, it cannot be excluded that, as in muscle, part of the transcriptional effects on T3 target genes, such as CPT1a and CPT2, in liver during food withdrawal/exercise may be due to increased local T3 levels.

In line with recent data obtained in mice at thermoneutrality (Raun et al., 2019), we found that exercise alone did not cause a significant response of the rat microbiome at thermoneutrality. Regarding the food withdrawal‐related compositional changes in the stool microbiome, at the genus level, most upregulated are Peptoclostridium, associated with ketone body synthesis and degradation, and Parabacteroides, associated with anti‐inflammatory effects. Of the downregulated genera, Lachnospiraceae are the most prominent. These changes, like those we observed in ketone body profiles, are reminiscent to those induced by ketogenic diets, and are associated with the reduction of epileptic seizures (Olson et al., 2018).

This study highlights altered responses to exercise and food withdrawal at thermoneutrality compared to lower temperatures: the effects of endurance exercise on both microbiota composition (Raun et al., 2019) and muscle BDNF expression (Sleiman et al., 2016), AMPK phosphorylation, and oxidative phenotype (Pedersen et al., 2009) are not encountered with mild exercise alone at thermoneutrality, a condition that we also observed to alter the effect of food withdrawal alone on transcription of thyroid hormone transporter MCT10 in liver (Schutkowski et al., 2014). Importantly, results of this study show that the combination of exercise with food withdrawal at thermoneutrality rescues the mild metabolic effect of exercise obtaining a strong oxidative response reminiscent of that of exercise alone at lower temperatures. The experiments being carried out at thermoneutrality are relatively better translatable to humans and may have beneficial metabolic applications.

## CONFLICT OF INTEREST

The authors declare no conflict of interest.

## AUTHOR CONTRIBUTIONS

Contributions to conception and design, acquisition of data, and analysis and interpretation of data: A.G, G.d.P, R.Si., M.C, M.R., W.B., R.K., A.G.U., A.S., R.Se, F.C., E.S., S.I., A.Lo., M.M., F.G., A.La., P.d.L.; drafting the article or revising it critically for important intellectual content: A.G., G.d.P, M.C., M.R., W.B., R.K., M.M., A.Lo., F.G., A.La., P.d.L.; final editing of the manuscript: F.G., A.La., P.d.L.; final approval of the version to be published: A.G, G.d.P, R.Si., M.C, M.R., W.B., R.K., A.G.U., A.S., R.Se, F.C., E.S., S.I., A.Lo., M.M., F.G., A.La., P.d.L.

## References

[phy214354-bib-0001] Aguer, C. , McCoin, C. S. , Knotts, T. A. , Thrush, A. B. , Ono‐Moore, K. , McPherson, R. , … Harper, M.‐E. (2015). Acylcarnitines: Potential implications for skeletal muscle insulin resistance. The FASEB Journal, 29, 336–345. 10.1096/fj.14-255901 25342132 PMC4285541

[phy214354-bib-0002] Aldiss, P. , Lewis, J. E. , Lupini, I. , Boocock, D. J. , Miles, A. K. , Ebling, F. J. P. , … Symonds, M. E. (2019). Exercise does not induce browning of WAT at thermoneutrality and induces an oxidative, myogenic signature in BAT. bioRxiv. 10.1101/649061 PMC7099615 32265830

[phy214354-bib-0003] Bocco, B. M. , Luzada, R. A. , Silvestre, D. H. , Santos, M. C. , Anne‐Palmer, E. , Rangel, I. F. , … Werneck‐de‐Castro, J. P. (2016). Thyroid hormone activation by type 2 deiodinase mediates exercise‐induced peroxisome proliferator‐activated receptor‐γ coactivator‐1α expression in skeletal muscle. Journal of Physiology, 594, 5255–5269. 27302464 10.1113/JP272440 PMC5023700

[phy214354-bib-0004] Brockhoff, M. , Rion, N. , Chojnowska, K. , Wiktorowicz, T. , Eickhorst, C. , Erne, B. , … Castets, P. (2017). Targeting deregulated AMPK/mTORC1 pathways improves muscle function in myotonic dystrophy type I. Journal of Clinical Investigation, 127, 549–563. 10.1172/JCI89616 28067669 PMC5272183

[phy214354-bib-0005] Caporaso, J. G. , Kuczynski, J. , Stombaugh, J. , Bittinger, K. , Bushman, F. D. , Costello, E. K. , … Knight, R. (2010). QIIME allows analysis of high‐throughput community sequencing data. Nature Methods, 7(5), 335–336. 10.1038/nmeth.f.303 20383131 PMC3156573

[phy214354-bib-0006] De Bock, K. , Richter, E. A. , Russell, A. P. , Eijnde, B. O. , Derave, W. , Ramaekers, M. , … Hespel, P. (2005). Exercise in the fasted state facilitates fibre type‐specific intramyocellular lipid breakdown and stimulates glycogen resynthesis in humans. Journal of Physiology, 564, 649–660. 10.1113/jphysiol.2005.083170 15705646 PMC1464435

[phy214354-bib-0007] De Lange, P. , Farina, P. , Moreno, M. , Ragni, M. , Lombardi, A. , Silvestri, E. , … Goglia, F. (2006). Sequential changes in the signal transduction responses of skeletal muscle following food deprivation. The FASEB Journal, 20(2579–2581), 3431–3441. 10.1096/fj.06-6025fje 17065218

[phy214354-bib-0008] De Lange, P. , Lanni, A. , Beneduce, L. , Moreno, M. , Lombardi, A. , Silvestri, E. , & Goglia, F. (2001). Uncoupling protein‐3 is a molecular determinant for the regulation of resting metabolic rate by thyroid hormone. Endocrinology, 142, 3414–3420. 10.1210/endo.142.8.8303 11459785

[phy214354-bib-0009] De Lange, P. , Moreno, M. , Silvestri, E. , Lombardi, A. , Goglia, F. , & Lanni, A. (2007). Fuel Economy in food‐deprived skeletal muscle. Signaling pathways and regulatory mechanisms. The FASEB Journal, 21, 3431–3441. 10.1096/fj.07-8527rev 17595346

[phy214354-bib-0010] De lange, P. , Ragni, M. , Silvestri, E. , Moreno, M. , Schiavo, L. , Lombardi, A. , … Lanni, A. (2004). Combined cDNA array/RT‐PCR analysis of gene expression profile in rat gastrocnemius muscle: Relation to its adaptive function in energy metabolism during fasting. The FASEB Journal, 18, 350–352. 10.1096/fj.03-0342fje 14656997

[phy214354-bib-0011] de Lange, P. , Senese, R. , Cioffi, F. , Moreno, M. , Lombardi, A. , Silvestri, E. , … Lanni, A. (2008). Rapid activation by 3,5,3‐L‐Triiodothyronine of adenosine 5 monophosphate activated protein kinase, acetyl‐coenzyme A carboxylase and Akt/Protein KinaseB signaling pathways: Relation to changes in fuel metabolism and myosin heavy‐chain protein content in rat gastrocnemius muscle in vivo. Endocrinology, 149, 6462–6470. 10.1210/en.2008-0202 18703632

[phy214354-bib-0012] Edgar, R. C. (2013). UPARSE: Highly accurate OTU sequences from microbial amplicon reads. Nature Methods, 10, 996–998. 23955772 10.1038/nmeth.2604

[phy214354-bib-0013] Edwards, J. G. , Bahl, J. J. , Flink, I. L. , Cheng, S. Y. , & Morkin, E. (1994). Thyroid hormone influences beta myosin heavy chain (beta MHC) expression. Biochemical and Biophysical Research Communications, 199, 1482–1488. 8147894 10.1006/bbrc.1994.1398

[phy214354-bib-0014] Fadrosh, D. W. , Ma, B. , Gajer, P. , Sengamalay, N. , Ott, S. , Brotman, R. M. , & Ravel, J. (2014). An improved dual‐indexing approach for multiplexed 16S rRNA gene sequencing on the Illumina MiSeq platform. Microbiome, 2, 6 10.1186/2049-2618-2-6 24558975 PMC3940169

[phy214354-bib-0015] Fischer, A. W. , Cannon, B. , & Nedergaard, J. (2019). The answer to the question "What is the best housing temperature to translate mouse experiments to humans?" is: Thermoneutrality. Molecular Metabolism, 26, 1–3. 10.1016/j.molmet.2019.05.006 31155502 PMC6667698

[phy214354-bib-0016] Giacco, A. , Delli Paoli, G. , Senese, R. , Cioffi, F. , Silvestri, E. , Moreno, M. , … de Lange, P. (2019). The saturation degree of fatty acids and their derived acylcarnitines determines the direct effect of metabolically active thyroid hormones on insulin sensitivity in skeletal muscle cells. The FASEB Journal, 33, 1811–1823. 10.1096/fj.201800724R 30204501

[phy214354-bib-0017] Hack, A. , Busch, V. , Pascher, B. , Busch, R. , Bieger, I. , Gempel, K. , & Baumeister, F. A. (2006). Monitoring of ketogenic diet for carnitine metabolites by subcutaneous microdialysis. Pediatric Research, 60, 93–96. 16690958 10.1203/01.pdr.0000219479.95410.79

[phy214354-bib-0018] Haczeyni, F. , Barn, V. , Mridha, A. R. , Yeh, M. M. , Estevez, E. , Febbraio, M. A. , … Farrell, G. C. (2015). Exercise improves adipose function and inflammation and ameliorates fatty liver disease in obese diabetic mice. Obesity (Silver Spring), 23, 1845–1855. 10.1002/oby.21170 26250514

[phy214354-bib-0019] Jaspers, R. T. , Zillikens, M. C. , Friesema, E. C. H. , delli Paoli, G. , Bloch, W. , Uitterlinden, A. G. , … de Lange, P. (2017). Exercise, fasting, and mimetics: Toward beneficial combinations? The FASEB Journal, 31, 14–28. 10.1096/fj.201600652R 27729415

[phy214354-bib-0020] Kosinski, C. , & Jornayvaz, F. R. (2017). Effects of ketogenic diets on cardiovascular risk factors: Evidence from animal and human studies. Nutrients, 9, 517 10.3390/nu9050517 PMC5452247 28534852

[phy214354-bib-0021] Lombardi, A. , Busiello, R. A. , De Matteis, R. , Lionetti, L. , Savarese, S. , Moreno, M. , … Goglia, F. (2019). Absence of uncoupling protein‐3 at thermoneutrality impacts lipid handling and energy homeostasis in mice. Cells, 8(8), 916 10.3390/cells8080916 PMC6721699 31426456

[phy214354-bib-0022] Marosi, K. , Moehl, K. , Navas‐Enamorado, I. , Mitchell, S. J. , Zhang, Y. , Lehrmann, E. , … Mattson, M. P. (2018). Metabolic and molecular framework for the enhancement of endurance by intermittent food deprivation. The FASEB Journal, 32, 3844–3858. 10.1096/fj.201701378RR 29485903 PMC5998977

[phy214354-bib-0023] Mathes, S. , Vanmunster, M. , Bloch, W. , & Suhr, F. (2019). Evidence for skeletal muscle fiber type‐specific expressions of mechanosensors. Cellular and Molecular Life Sciences, 15, 2987–3004. 10.1007/s00018-019-03026-3 PMC11105595 30701284

[phy214354-bib-0024] McKie, G. L. , Medak, K. D. , Knuth, C. M. , Shamshoum, H. , Townsend, L. K. , Peppler, W. T. , & Wright, D. C. (2019). Housing temperature affects the acute and chronic metabolic adaptations to exercise in mice. Journal of Physiology, 597, 4581–4600. 10.1113/JP278221 31297830

[phy214354-bib-0025] Moreno, M. , Lombardi, A. , De Lange, P. , Silvestri, E. , Ragni, M. , Lanni, A. , & Goglia, F. (2003). Fasting, lipid metabolism, and triiodothyronine in rat gastrocnemius muscle: Interrelated roles of uncoupling protein 3, mitochondrial thioesterase, and coenzyme Q. The FASEB Journal, 9, 1112–1114. 10.1096/fj.02-0839fje 12692085

[phy214354-bib-0026] Oksanen, J. , Blanchet, F. G. , Friendly, M. , Kindt, R. , Legendre, P. , McGlinn, D. , … Wagner, H. (2013). Vegan: Community ecology package. Retrieved from http://CRAN.R-project.org/package=vegan.

[phy214354-bib-0027] Olson, C. A. , Vuong, H. E. , Yano, J. M. , Liang, Q. Y. , Nusbaum, D. J. , & Hsiao, E. Y. (2018). The gut microbiota mediates the anti‐seizure effects of the ketogenic diet. Cell, 173, 1728–1741. 10.1016/j.cell.2018.04.027 29804833 PMC6003870

[phy214354-bib-0028] Pedersen, B. K. , Pedersen, M. , Krabbe, K. S. , Bruunsgaard, H. , Matthews, V. B. , & Febbraio, M. A. (2009). Role of exercise‐induced brain‐derived neurotrophic factor production in the regulation of energy homeostasis in mammals. Experimental Physiology, 94, 1153–1160. 19748969 10.1113/expphysiol.2009.048561

[phy214354-bib-0029] Quast, C. , Pruesse, E. , Yilmaz, P. , Gerken, J. , Schweer, T. , Yarza, P. , … Glöckner, F. O. (2012). The SILVA ribosomal RNA gene database project: Improved data processing and web‐based tools. Nucleic Acids Research, 41, D590–D596. 10.1093/nar/gks1219 23193283 PMC3531112

[phy214354-bib-0030] R Core Team . (2017). A language and environment for statistical computing. Vienna, Austria: R Foundation for Statistical Computing Retrieved from https://www.R-project.org/.

[phy214354-bib-0031] Raun, S. H. , Olguín, C. H. , Karavaeva, I. , Ali, M. , Møller, L. L. V. , Kot, W. , … Sylow, L. (2019). Housing temperature influences exercise training adaptations in mice. bioRxiv, 10.1101/651588 PMC7096511 32214091

[phy214354-bib-0032] Rossi, A. , Ruoppolo, M. , Formisano, P. , Villani, G. , Albano, L. , Gallo, G. , … Melis, D. (2018). Insulin‐resistance in glycogen storage disease type Ia: Linking carbohydrates and mitochondria? J. Inherit. Metab., 41, 985–995. 10.1007/s10545-018-0149-4 29435782

[phy214354-bib-0033] Ruoppolo, M. , Campesi, I. , Scolamiero, E. , Pecce, R. , Caterino, M. , Cherchi, S. , … Franconi, F. (2014). Serum metabolomic profiles suggest influence of sex and oral contraceptive use. American Journal of Translational Research, 6, 614–624. 25360225 PMC4212935

[phy214354-bib-0034] Ruoppolo, M. , Caterino, M. , Albano, L. , Pecce, R. , Di Girolamo, M. G. , Crisci, D. , … Campesi, I. (2018). Targeted metabolomic profiling in rat tissues reveals sex differences. Scientific Reports, 8, 4663. 29549307 10.1038/s41598-018-22869-7 PMC5856765

[phy214354-bib-0035] Ruoppolo, M. , Scolamiero, E. , Caterino, M. , Mirisola, V. , Franconi, F. , & Campesi, I. (2015). Female and male human babies have distinct blood metabolomic patterns. Molecular BioSystems, 11, 2483–2492. 10.1039/C5MB00297D 26140445

[phy214354-bib-0036] Schutkowski, A. , Wege, N. , Stangl, G. I. , & König, B. (2014). Tissue‐specific expression of monocarboxylate transporters during fasting in mice. PLoS ONE, 9, e112118 10.1371/journal.pone.0112118 25390336 PMC4229183

[phy214354-bib-0037] Scolamiero, E. , Villani, G. R. , Ingenito, L. , Pecce, R. , Albano, L. , Caterino, M. , … Ruoppolo, M. (2014). Maternal vitamin B12 deficiency detected in expanded newborn screening. Clinical Biochemistry, 47, 312–317. 25204964 10.1016/j.clinbiochem.2014.08.020

[phy214354-bib-0038] Senese, R. , Valli, V. , Moreno, M. , Lombardi, A. , Busiello, R. A. , Cioffi, F. , … de Lange, P. (2011). Uncoupling protein 3 expression levels influence insulin sensitivity, fatty acid oxidation, and related signaling pathways. Pflugers Archiv ‐ European Journal of Physiology, 461, 153–164. 10.1007/s00424-010-0892-3 21058020

[phy214354-bib-0039] Sleiman, S. F. , Henry, J. , Al‐Haddad, R. , El Hayek, L. , Abou Haidar, E. , Stringer, T. , … Chao, M. V. (2016). Exercise promotes the expression of brain derived neurotrophic factor (BDNF) through the action of the ketone body β‐hydroxybutyrate. Elife, e15092. 27253067 10.7554/eLife.15092 PMC4915811

[phy214354-bib-0040] Svensson, K. , Albert, V. , Cardel, B. , Salatino, S. , & Handschin, C. (2016). Skeletal muscle PGC‐1a modulates systemic ketone body homeostasis and ameliorates diabetic hyperketonemia in mice. The FASEB Journal, 30, 1976–1986. 26849960 10.1096/fj.201500128 PMC4970654

[phy214354-bib-0041] Van Proeyen, K. , Szlufcik, K. , Nielens, H. , Ramaekers, M. , & Hespel, P. (1985). (2011) Beneficial metabolic adaptations due to endurance exercise training in the fasted state. Journal of Applied Physiology, 110, 236–245. 10.1152/japplphysiol.00907.2010 PMC3253005 21051570

[phy214354-bib-0042] Wang, Q. , Garrity, G. M. , Tiedje, J. M. , & Cole, J. R. (2007). Naive Bayesian classifier for rapid assignment of rRNA sequences into the new bacterial taxonomy. Applied and Environment Microbiology, 73, 5261–5267. 10.1128/AEM.00062-07 PMC1950982 17586664

[phy214354-bib-0043] Yu, T. , Chang, Y. , Gao, X. L. , Li, H. , & Zhao, P. (2017). Dynamic expression and the role of BDNF in exercise‐induced skeletal muscle regeneration. International Journal of Sports Medicine, 38, 959–966. 28965341 10.1055/s-0043-118343

[phy214354-bib-0044] Zheng, D. M. , Bian, Z. , Furuya, N. , Oliva Trejo, J. A. , Takeda‐Ezaki, M. , Takahashi, K. , … Ezaki, J. (2015). A treadmill exercise reactivates the signaling of the mammalian target of rapamycin (mTor) in the skeletal muscles of starved mice. Biochemical and Biophysical Research Communications, 456, 519–526. 25485704 10.1016/j.bbrc.2014.11.118

