# Supplementary figures and images for: Exercise with food withdrawal at thermoneutrality impacts fuel use, the microbiome, AMPK phosphorylation, muscle fibers, and thyroid hormone levels in rats
Source: Physiol Rep. 2020 Feb 7;8(3):e14354. doi: 10.14814/phy2.14354 (PMC7007447; doi:10.14814/phy2.14354)

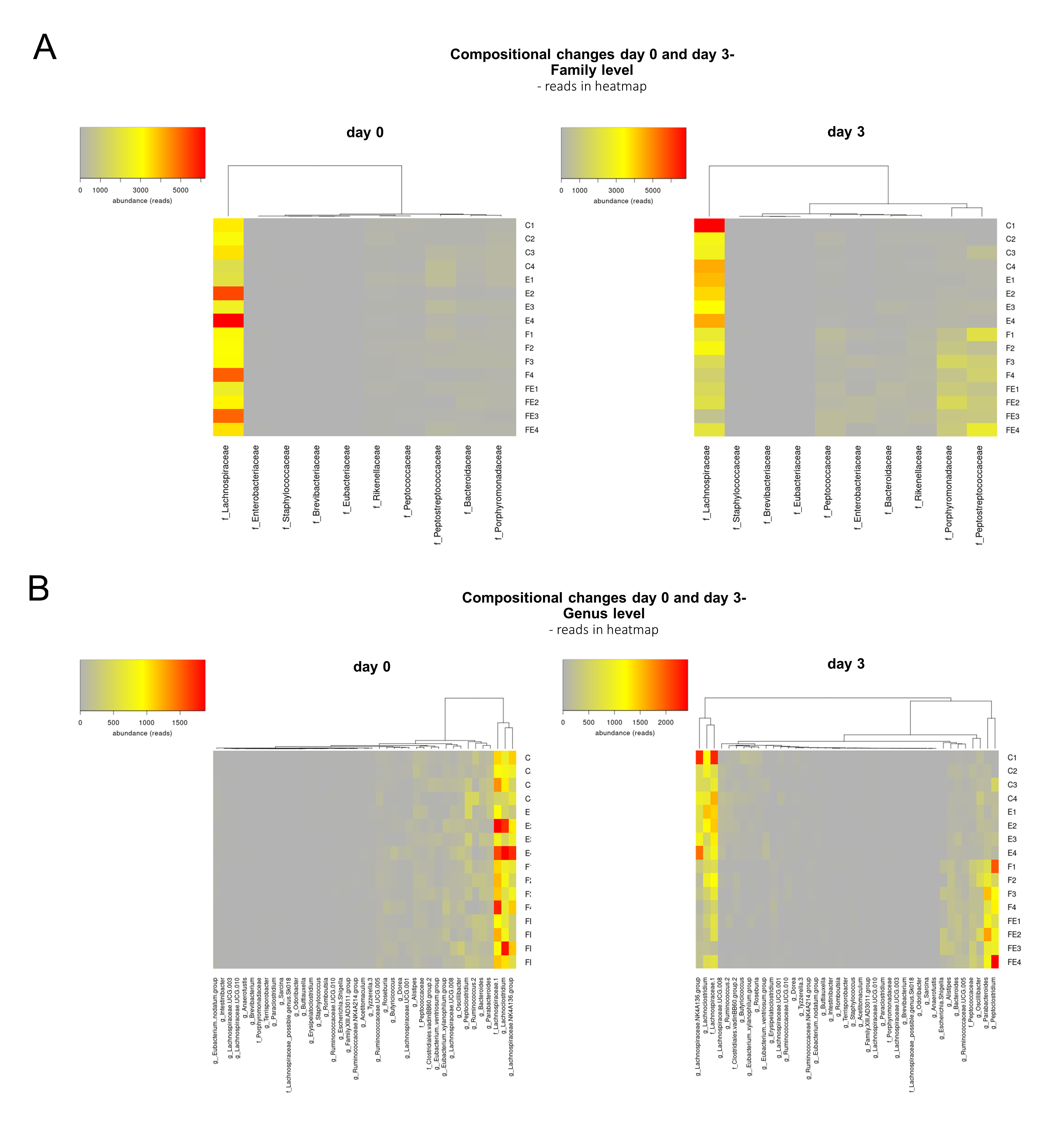

Supplement: Supplementary file 1 [file PHY2-8-e14354-s001.tif]
