# Supplementary material for: Exercise with food withdrawal at thermoneutrality impacts fuel use, the microbiome, AMPK phosphorylation, muscle fibers, and thyroid hormone levels in rats
Source: Physiol Rep. 2020 Feb 7;8(3):e14354. doi: 10.14814/phy2.14354 (PMC7007447; doi:10.14814/phy2.14354)
Supplement: Supplementary file 2 [file PHY2-8-e14354-s002.docx]

**Figure S1. Effect of food withdrawal and exercise (FE) on stool microbiome composition**. A: Absolute composition (day 0, left panel, day 3, right panel ) in reads at family-level. Only families that were significantly different between the groups are presented. B: As A, at genus-level. Abbreviations: C = chow-fed controls, E= chow-fed exercised, F = food withdrawn, FE = food withdrawn and exercised.
